# A Phosphorylation‐Dependent Partner‐Switching‐Like Module Regulates a Glycosyltransferase Required for Heterocyst Polysaccharide Layer Formation in *Anabaena* sp. Strain PCC 7120

**DOI:** 10.1111/mmi.70091

**Published:** 2026-06-22

**Authors:** Mai Harada, Satoshi Matsuoka, Shigeki Ehira

**Affiliations:** ^1^ Department of Biological Sciences, Graduate School of Science Tokyo Metropolitan University Hachioji Tokyo Japan; ^2^ Department of Biochemistry and Molecular Biology, Graduate School of Science and Engineering Saitama University Saitama Saitama Japan

## Abstract

Heterocyst‐specific polysaccharide (Hep) is an essential component of the heterocyst envelope in the filamentous cyanobacterium *Anabaena* sp. PCC 7120; however, the regulatory mechanisms that control Hep layer formation remain poorly understood. Herein, we reveal that Hep layer formation is regulated by a phosphorylation‐dependent partner‐switching‐like system involving the phosphatase HenR and the STAS domain‐containing glycosyltransferase All4160. A nonphosphorylatable All4160 variant restores Hep formation in a *henR* disruptant, indicating that phosphorylation negatively regulates All4160 function. Using a bacterial two‐hybrid assay, we identified two candidate kinases that interact with All4160, that is, Alr3423 and All2284, which phosphorylate All4160 in vitro. Genetic analysis revealed that deletion of alr3423, not all2284, suppresses the defect of the *henR* disruptant, indicating that Alr3423 plays a key role in regulating All4160 in vivo. This study identifies a phosphorylation switch that controls Hep layer formation and reveals a regulatory mechanism acting directly on a polysaccharide biosynthetic enzyme.

## Graphical abstract

The glycosyltransferase All4160 is regulated by the reversible phosphorylation of Ser74. The kinase Alr3423 phosphorylates and inactivates All4160, while the SpoIIE‐like phosphatase HenR restores its activity through dephosphorylation. A nonphosphorylatable variant of All4160 bypasses the need for HenR, which demonstrates that a phosphorylation‐dependent partner‐switching‐like module directly controls enzyme activity.

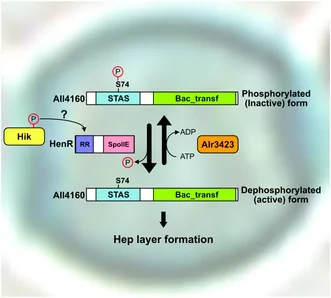

## 1 Introduction

Cyanobacteria are a phylogenetically and physiologically diverse group of bacteria that can perform oxygenic photosynthesis and play central roles in global carbon and nitrogen cycles. They function as major primary producers in many ecosystems by fixing carbon dioxide and, in some species, dinitrogen. Because nitrogenase, the enzyme responsible for nitrogen fixation, is highly sensitive to oxygen, cyanobacteria have evolved distinct strategies to protect this activity from oxygen‐induced inactivation (Berman‐Frank et al. 2003). Unicellular cyanobacteria temporally separate photosynthesis and nitrogen fixation, restricting nitrogen fixation to the night. Meanwhile, many filamentous cyanobacteria spatially separate these processes. The filamentous cyanobacterium *Anabaena* sp. strain PCC 7120 (hereinafter referred to as *Anabaena*) differentiates cells called heterocysts, which are specialized for nitrogen fixation, at semi‐regular intervals along its filament under nitrogen‐deprived conditions (Kumar et al. 2010; Flores et al. 2019). Heterocysts provide a microoxic intracellular environment that supports nitrogenase activity via multiple coordinated mechanisms. During differentiation, the oxygen‐evolving photosystem II is inactivated, whereas respiration is activated to consume residual oxygen. In addition, heterocysts are surrounded by a multilayered envelope consisting of heterocyst‐specific glycolipid and polysaccharide layers (Hgl and Hep layers, respectively), which function as a barrier to oxygen diffusion. The appropriate formation of the Hep layer is essential for the assembly and function of the heterocyst envelope.

The synthesis of the Hep layer depends on the HEP island, a cluster of 17 genes (alr2825–alr2841) that are induced under nitrogen‐deprived conditions (Ehira et al. 2003; Huang et al. 2005). In addition, several genes that encode glycosyltransferases and are located outside the HEP island, such as *hepB*, alr3699, and all4160, are involved in Hep layer formation (Wang et al. 2007). Other genes implicated in Hep layer formation are genes with regulatory functions, including those encoding HepK and HepN, which are histidine kinases that comprise a two‐component regulatory system, HepS, a serine/threonine kinase, and HenR, a serine phosphatase (Zhu et al. 1998; Ning and Xu 2004; Fan et al. 2006). However, despite these advances in the knowledge of the Hep layer, the regulatory mechanisms underlying its biosynthesis remain poorly understood.

One potential regulatory mechanism of Hep layer formation involves reversible protein phosphorylation. Specifically, in the partner‐switching system (PSS)—a regulatory mechanism that controls gene expression in response to environmental stimuli and comprises a serine kinase, a SpoIIE‐like serine phosphatase, and a protein containing the STAS domain (Bouillet et al. 2018)—the activity of sigma factors is regulated by the phosphorylation state of the STAS protein, which is in turn controlled by the kinase and the phosphatase. Although PSS‐like regulatory systems have been identified in various bacteria, their role in cyanobacteria remains unclear. For instance, although the Hmp and Hcy PSS‐like systems in *Nostoc punctiforme* are known to regulate hormogonium development and motility (Riley et al. 2018; Klicki et al. 2022), the molecular mechanisms underlying this regulation still need clarification. Notably, All4160 contains an N‐terminal STAS domain, and HenR is considered a SpoIIE‐like phosphatase. Accordingly, All4160 and HenR might constitute a PSS‐like regulatory module that controls Hep layer formation. In this study, we investigated the functional relationship between HenR and All4160, focusing on the role of the phosphorylation of the All4160 STAS domain in regulating Hep layer formation.

## 2 Results

### 2.1 Suppression of the Defects of the *henR* Disruptant by a Nonphosphorylatable All4160 Variant

**FIGURE 1:**
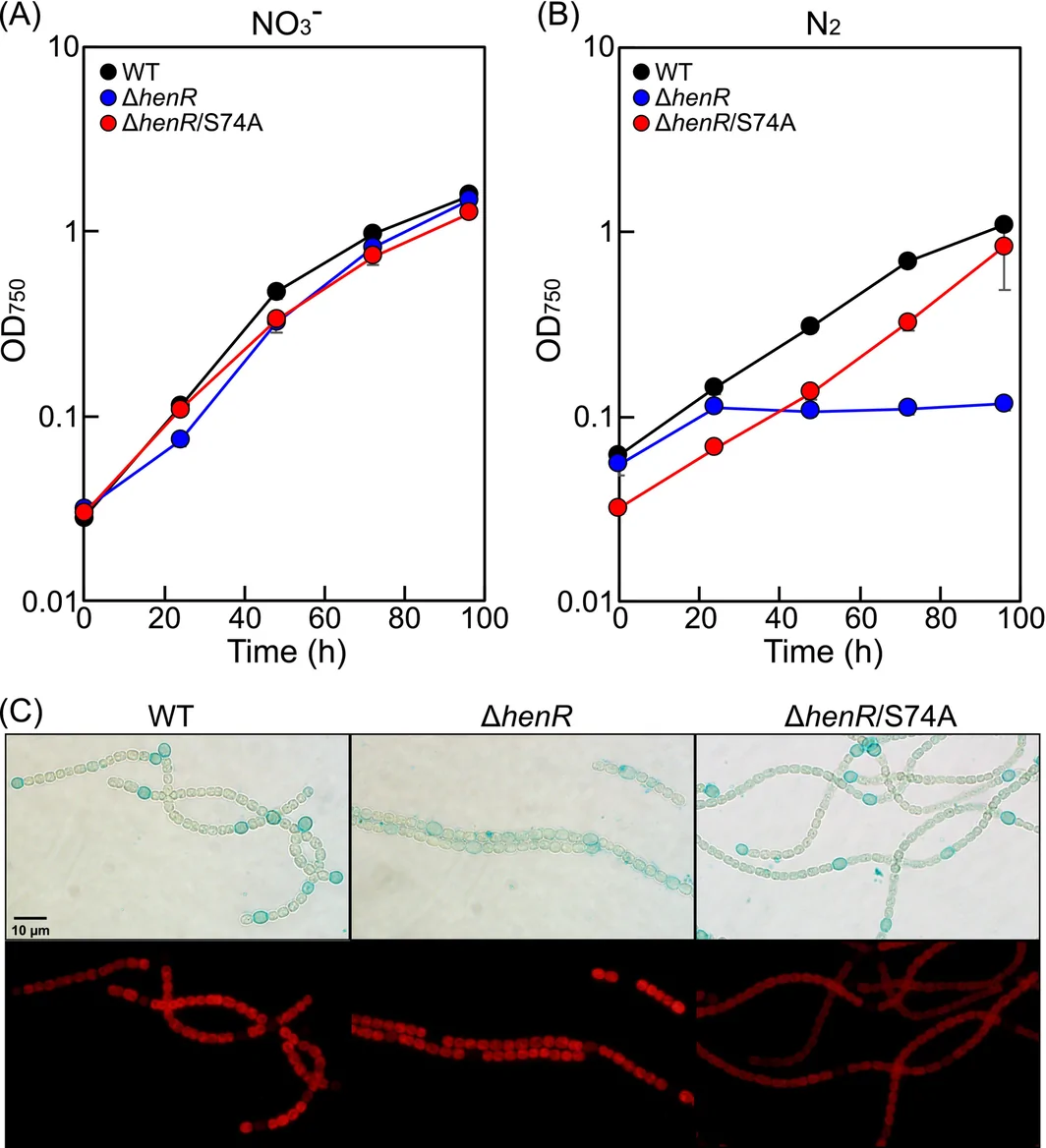
Restoration of Hep layer formation and nitrogen fixation by a nonphosphorylatable All4160 variant in the absence of HenR. Growth of the wild‐type strain (WT, black circles), the *henR* disruptant (Δ*henR*, blue circles), and Δ*henR* carrying a plasmid expressing an All4160 variant in which the phosphorylatable serine residue was replaced with alanine (Δ*henR*/S74A, red circles) with nitrate (A) or dinitrogen (B) as nitrogen sources. (C) Micrographs of Alcian blue‐stained cells of WT (left panels), Δ*henR* (center panels), and Δ*henR*/S74A (right panels). The upper panels show bright‐field images, and the lower panels show autofluorescence.

To investigate the functional relationship between HenR and All4160, we examined the effect of the phosphorylation state of All4160 on Hep layer formation and nitrogen fixation. As previously reported (Fan et al. 2006), the *henR* disruptant (Δ*henR*) generated in this study showed impaired growth under nitrogen‐fixing conditions, whereas growth was comparable to that of the wild‐type strain in the presence of nitrate (Figure 1A,B). Consistently, Alcian blue staining revealed that Δ*henR* failed to form an appropriate Hep layer (Figure 1C). Similar to Δ*henR*, the All4160 disruptant (Δ4160) also exhibited impaired diazotrophic growth and defective Hep layer formation (Figure S1) (Wang et al. 2007). To assess the role of the phosphorylation of the STAS domain of All4160, we generated Δ*henR*/S74A, a Δ*henR* strain carrying a plasmid that expresses an All4160 variant containing alanine in place of the putative phosphorylation site (Ser74) (Figure S2). The Δ*henR*/S74A strain restored growth under nitrogen‐fixing conditions and formed heterocysts with a Hep layer detectable upon staining with Alcian blue (Figure 1B,C). In contrast, expression of wild‐type All4160 in the Δ*henR* background did not restore diazotrophic growth (Figure S3). These results indicate that the phosphorylation of the STAS domain negatively regulates All4160 activity in Hep layer formation.

### 2.2 Identification of Kinases That Phosphorylate the All4160 STAS Domain

**FIGURE 2:**
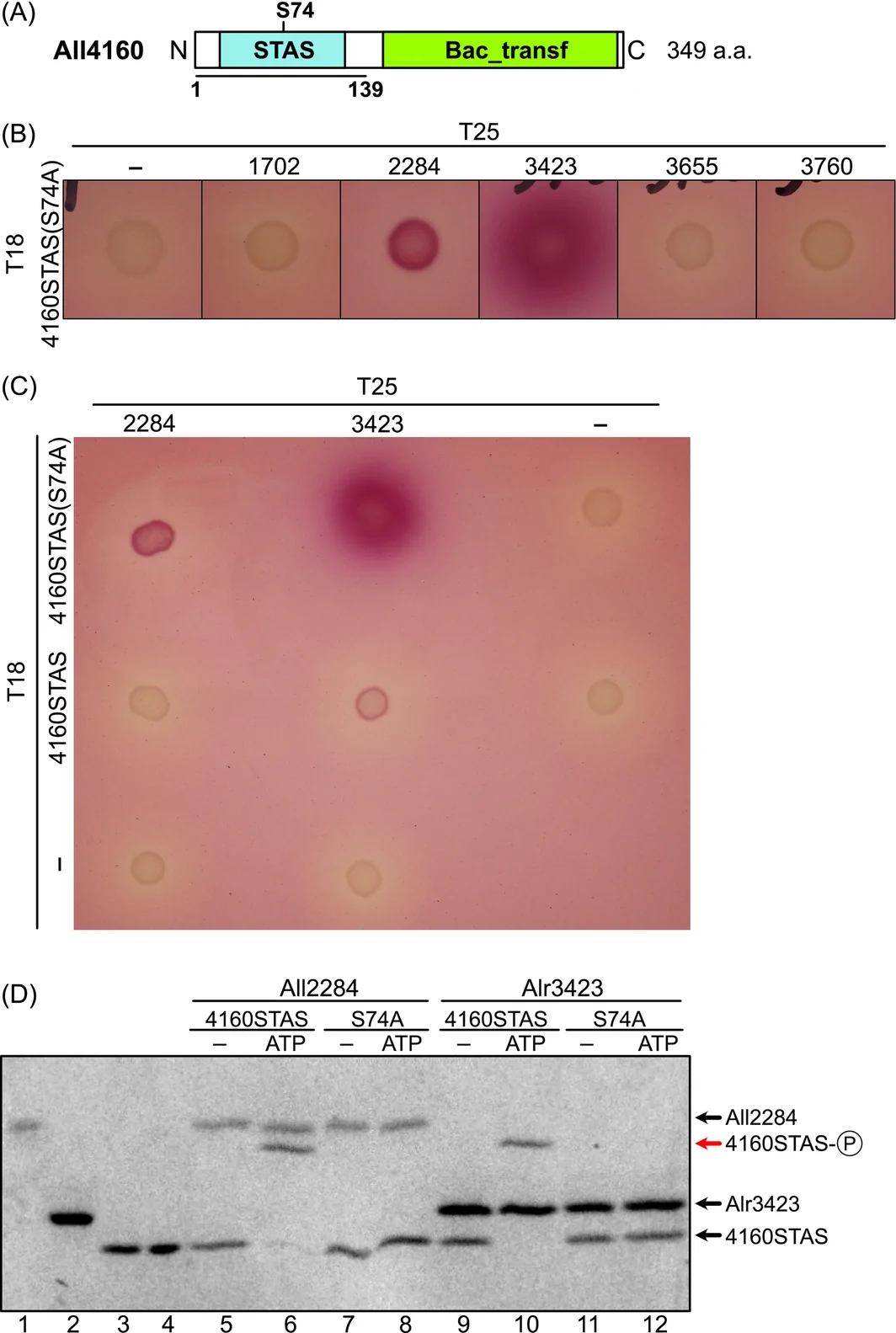
Phosphorylation of the All4160 STAS domain by All2284 and Alr3423. (A) Domain structure of All4160. The protein contains an N‐terminal STAS domain and a C‐terminal Bacterial sugar transferase (Bac_transf) domain. S74 indicates the putative phosphorylation site. The STAS region (residues 1–139) was used in the bacterial two‐hybrid (BACTH) assay. (B) Screening for kinases interacting with the All4160 STAS domain (S74A variant). The STAS domain of All4160 carrying the S74A substitution (4160STAS(S74A)) was fused to the T18 fragment of adenylate cyclase, and candidate kinases (All1702, All2284, Alr3423, Alr3655, or Alr3760) were fused to the T25 fragment. The plasmids were cotransformed into *Escherichia coli* , and interactions were detected on MacConkey agar containing maltose. “–” indicates plasmids expressing only the T18 or T25 fragments. (C) Interaction of the wild‐type STAS domain with All2284 and Alr3423. (D) In vitro phosphorylation assay analyzed by Phos‐tag SDS‐PAGE. Recombinant proteins were incubated with or without ATP prior to electrophoresis. Proteins included in each lane were as follows: Lane 1, All2284; lane 2, Alr3423; lane 3, the All4160 STAS domain (4160STAS); lane 4, the S74A variant of the STAS domain (S74A); lanes 5–8, All2284 with 4160STAS or S74A; lanes 9–12, Alr3423 with 4160STAS or S74A. ATP was added to lanes 6, 8, 10, and 12.

Next, we sought to identify kinases responsible for the phosphorylation of the All4160 STAS domain. The *Anabaena* genome encodes five serine kinase candidates, that is, All1702, All2284, Alr3423, Alr3655, and Alr3760. To test the interaction between these kinases and the All4160 STAS domain, we performed a bacterial adenylate cyclase two‐hybrid (BACTH) assay. Because phosphorylation of the conserved serine residue in the STAS domain weakens its interaction with kinases (Yang et al. 1996), we used a nonphosphorylatable variant, 4160STAS(S74A), in which the putative phosphorylation site was replaced with alanine (Figure 2A). Qualitative analysis on MacConkey agar showed that All2284 and Alr3423 interacted with 4160STAS(S74A) (Figure 2B). These interactions were stronger than those of both kinases with the wild‐type STAS domain (4160STAS), suggesting that these kinases phosphorylate the Ser74 residue of the All4160 STAS domain (Figure 2C).

To determine whether All2284 and Alr3423 phosphorylate the All4160 STAS domain, we performed in vitro phosphorylation assays. The recombinant proteins All2284, Alr3423, 4160STAS, and 4160STAS(S74A) were expressed as His‐tagged proteins and purified by affinity chromatography (Figure S4). All2284 and Alr3423 were separately incubated with 4160STAS in the presence or absence of ATP, and the proteins were analyzed by Phos‐tag SDS‐PAGE, in which phosphorylated proteins migrated more slowly than their nonphosphorylated counterparts. When All2284 was incubated with 4160STAS in the presence of ATP, a mobility shift of 4160STAS was observed, whereas no shift was detected in the absence of ATP (Figure 2D, lanes 5 and 6). In contrast, incubation of All2284 with 4160STAS(S74A) did not cause a band shift even in the presence of ATP (Figure 2D, lanes 7 and 8). Similar results were obtained with Alr3423 (Figure 2D, lanes 9–12). These results indicate that both kinases directly phosphorylate the Ser74 residue of the All4160 STAS domain in an ATP‐dependent manner.

**FIGURE 3:**
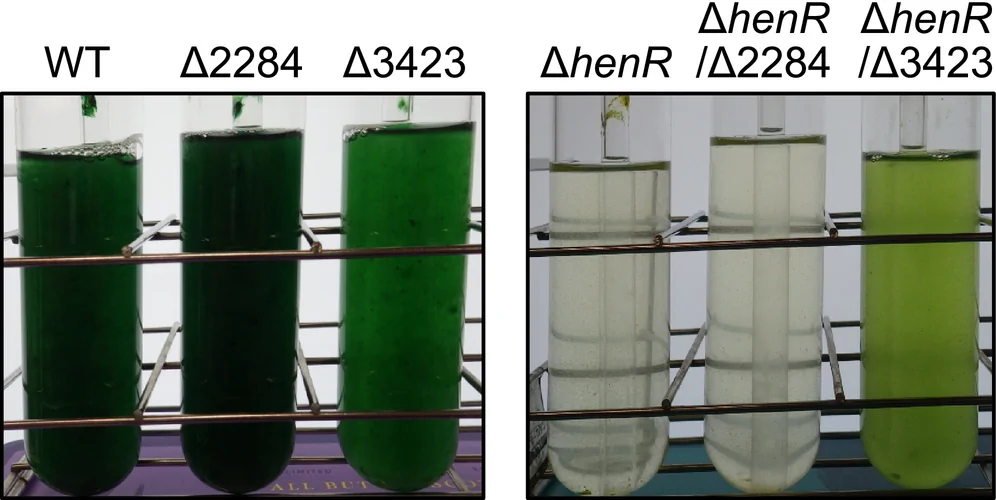
Genetic interaction between *henR* and candidate kinase genes all2284 and alr3423. Growth phenotypes of the indicated strains after 7 days of cultivation under nitrogen‐fixing conditions. Representative culture tubes are shown. Strains included the wild‐type strain (WT), single disruptants (Δ2284 and Δ3423), Δ*henR*, and double disruptants (Δ*henR*/Δ2284 and Δ*henR*/Δ3423).

### 2.3 Genetic Interactions Between *henR* and Candidate Kinase Genes

Having established that All2284 and Alr3423 phosphorylate the All4160 STAS domain in vitro (Figure 2), we then investigated the effect of these kinases on All4160 function in vivo. Because a nonphosphorylatable All4160 variant suppresses the defects of Δ*henR* (Figure 1), we hypothesized that loss of the kinase activity responsible for All4160 phosphorylation would also suppress the Δ*henR* phenotype. To test this theory, we generated double disruptants in which both *henR* and either all2284 or alr3423 were disrupted. As shown in Figure 3, single disruptants of all2284 (Δ2284) and alr3423 (Δ3423) grew under nitrogen‐fixing conditions. Meanwhile, the Δ*henR*/Δ2284 double disruptant exhibited impaired growth similar to that of Δ*henR*, whereas disruption of alr3423 in the Δ*henR* background restored diazotrophic growth (Figure 3). These results indicate that Alr3423, but not All2284, is functionally involved in regulating All4160 activity during Hep layer formation.

## 3 Discussion

**FIGURE 4:**
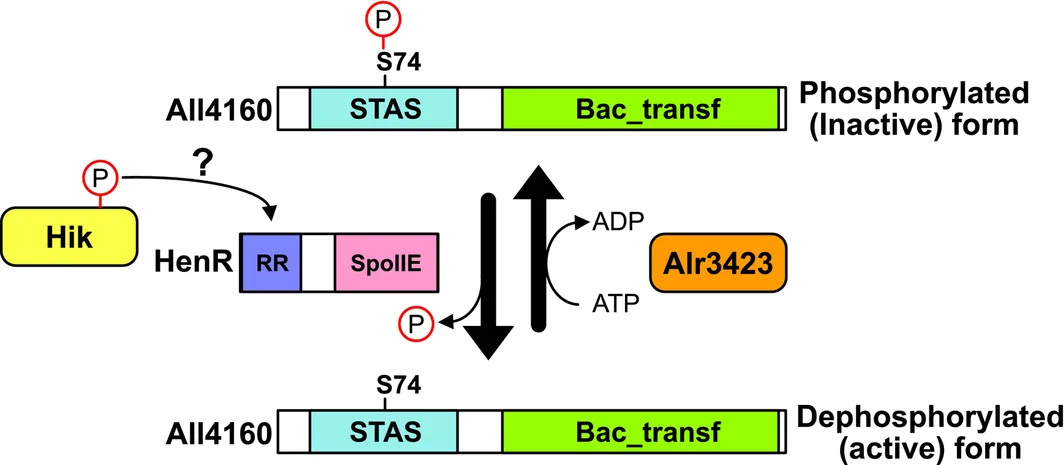
Phosphorylation‐dependent regulation of All4160 activity. All4160 activity is probably regulated by the phosphorylation and dephosphorylation of its STAS domain, which is catalyzed by Alr3423 and HenR, respectively. HenR contains an N‐terminal response regulator receiver (RR) domain and a C‐terminal SpoIIE domain. HenR activity may be regulated by a cognate histidine kinase (Hik).

In this study, we identified a phosphorylation‐dependent regulatory module that controls the Hep layer formation during heterocyst differentiation in *Anabaena*. Our results suggest that All4160 activity is modulated via the antagonistic actions of a kinase and a phosphatase, as observed in canonical PSSs. However, unlike canonical PSSs, which regulate gene expression via sigma factors, this regulatory module appears to control the activity of a polysaccharide synthetic enzyme. The STAS domain‐containing glycosyltransferase All4160 is negatively regulated by phosphorylation by Alr3423, and the SpoIIE‐like phosphatase HenR is required for its activation (Figure 4). Consistent with this model, the nonphosphorylatable All4160 variant restores both Hep formation and diazotrophic growth in the absence of HenR (Figure 1). Additionally, a genetic interaction exists between *henR* and alr3423; disruption of alr3423 suppresses the defects of Δ*henR* (Figure 3). Although in vivo phosphorylation of All4160 remains to be directly demonstrated, the combined genetic and in vitro biochemical evidence strongly supports a phosphorylation‐dependent regulatory mechanism controlling Hep layer formation.

HenR contains an N‐terminal receiver domain characteristic of response regulators, suggesting that its phosphatase activity may be regulated by a cognate histidine kinase (Hik) (Figure 4). This suggests that Hep layer formation is controlled by the modulation of HenR phosphatase activity through a hitherto unidentified Hik. Although the signals that regulate Hep layer formation have not been identified, the function of the heterocyst‐specific envelope as an oxygen diffusion barrier suggests that intracellular oxygen levels may serve as regulatory signals for this Hik. Identification of the cognate Hik and its regulatory signals is important for understanding the regulatory network that governs Hep layer formation during heterocyst differentiation.

Npu_F0312, an ortholog of Alr3423 in *N. punctiforme* , is not a member of the Hmp and Hcy systems, indicating that it may have a specific role in heterocyst differentiation. In contrast, the All2284 ortholog is absent from *N. punctiforme* , which is consistent with the dispensability of All2284 for Hep layer formation mediated by All4160 and HenR. The facts that the binding and phosphorylation activities of All2284 toward All4160 were lower than those of Alr3423 and that the all2285 gene, which encodes a glycosyltransferase with an N‐terminal STAS domain, is located adjacent to all2284 in the *Anabaena* genome (Kaneko et al. 2001) suggest that All2284 primarily acts on All2285 rather than on All4160 in vivo. Analysis using InterPro indicates that proteins containing both STAS and Bacterial sugar transferase domains are widely conserved among cyanobacteria but rarely found outside this lineage. An example of these proteins is XssP, which is essential for the biosynthesis of the sulfated exopolysaccharide synechan in *Synechocystis* sp. PCC 6803 (Maeda et al. 2021). These results indicate that the phosphorylation‐dependent regulation of polysaccharide biosynthetic enzymes is a lineage‐specific regulatory strategy.

## 4 Experimental Procedures

### 4.1 Strains and Growth Conditions

*Anabaena* sp. strain PCC 7120 and its derivatives were grown in BG11 medium or BG11_0_ medium (BG11 lacking combined nitrogen) at 30°C under continuous illumination (30 μmol photons m^−2^ s^−1^) provided by cool white fluorescent light. Liquid cultures were bubbled with air supplemented with 1.0% (v/v) CO_2_. For growth under nitrogen‐fixing conditions, cells grown in BG11 were washed and resuspended in BG11_0_ as previously described (Ehira and Ohmori 2006). When required, spectinomycin and streptomycin were added at final concentrations of 2.5 μg mL^−1^ each, and neomycin at 30 μg mL^−1^.

### 4.2 Mutant Construction

All strains used in this study are listed in Table S1. Transformation of *Anabaena* was conducted by triparental conjugation as previously reported (Elhai et al. 1997). For the construction of gene disruptants, the plasmid pRL271 (Black et al. 1993), which carries *sacB*, was used, and antibiotic‐ and sucrose‐resistant clones were selected to obtain double recombinants. Complete segregation was confirmed by PCR. To generate all4160‐expressing strains in the Δ*henR* background, a DNA fragment containing the upstream region and coding sequence of all4160 was cloned into the suicide vector pSU101 (Ehira et al. 2017), and single recombinants were selected based on neomycin resistance. A nonphosphorylatable variant of All4160 (S74A) was generated by site‐directed mutagenesis, in which the nucleotides at positions 720 and 721 (A and G) were replaced with G and C, respectively, using the PrimeSTAR Mutagenesis Basal Kit (Takara Bio Inc.).

### 4.3 BACTH Assay

Protein–protein interactions were analyzed using the BACTH system (Euromedex). A DNA fragment encoding the STAS domain (residues 1–139) of All4160 was amplified and cloned between the XbaI and SacI sites of pUT18C to generate an N‐terminal fusion with the T18 fragment of adenylate cyclase. The coding regions of candidate kinases, that is, All1702, All2284, Alr3423, Alr3655, and Alr3760, were amplified and cloned between the XbaI and EcoRI sites of pKT25 to generate N‐terminal fusions with the T25 fragment. The resulting plasmids were cotransformed into *Escherichia coli* BTH101. Transformants carrying both plasmids were grown in LB medium supplemented with 100 μg mL^−1^ ampicillin and 50 μg mL^−1^ kanamycin at 30°C. Aliquots (1 μL) of overnight cultures were spotted onto MacConkey agar plates supplemented with 1% (w/v) maltose, 0.5 mM IPTG, 100 μg mL^−1^ ampicillin, and 50 μg mL^−1^ kanamycin and incubated for 24 h at 30°C. Protein–protein interactions were evaluated by colony color development relative to positive and negative controls.

### 4.4 In Vitro Phosphorylation Assay

Purified STAS domain of All4160 (1 μg) or its S74A variant (1 μg) was incubated with All2284 (1 μg) or Alr3423 (1 μg) at 28°C for 30 min in phosphorylation buffer [25 mM Tris–HCl (pH 7.9), 50 mM NaCl, 5 mM MgCl_2_, and 1 mM dithiothreitol] in the presence or absence of 2 mM ATP. The reactions were terminated by adding 2× SDS‐PAGE sample buffer, and proteins were resolved on 15% SDS‐PAGE gels containing 75 μM Phos‐tag acrylamide (FUJIFILM Wako Pure Chemical Corporation) and 150 μM MnCl_2_. After electrophoresis, proteins were visualized using EzStain Aqua (ATTO Corporation).

## Author Contributions

**Mai Harada:** investigation, methodology, data curation, validation, formal analysis, visualization, writing – review and editing. **Satoshi Matsuoka:** resources, methodology, writing – review and editing. **Shigeki Ehira:** writing – review and editing, writing – original draft, conceptualization, funding acquisition, supervision, visualization, project administration.

## Funding

This work was supported by Japan Society for the Promotion of Science (JP22K19138 and JP25K01938) Japan Science and Technology Agency (JPMJCR25J3).

## Ethics Statement

The authors have nothing to report.

## Conflicts of Interest

The authors declare no conflicts of interest.

## Supporting information


**Table S1:** Strains used in this study.
**Figure S1:** Phenotypes of the all4160 mutant (Δ4160).
**Figure S2:** Multiple sequence alignment of STAS domain amino acid sequences.
**Figure S3:** Expression of wild‐type All4160 does not restore the nitrogen‐fixation defect of Δ*henR*.
**Figure S4:** Recombinant proteins used for the phosphorylation assay.

## Data Availability

The data that support the findings of this study are available from the corresponding author upon reasonable request.
