# Supplementary material for: A Phosphorylation‐Dependent Partner‐Switching‐Like Module Regulates a Glycosyltransferase Required for Heterocyst Polysaccharide Layer Formation in Anabaena sp. Strain PCC 7120
Source: Mol Microbiol. 2026 Jun 22;126(3):237–43. doi: 10.1111/mmi.70091 (PMC13555669; doi:10.1111/mmi.70091)
Supplement: Supplementary file 1 [file MMI-126-237-s001.pdf]

Table S1. Strains used in this study

Strains	Description
WT	*Anabaena* sp. strain PCC 7120
Δ*henR*	The *henR* coding region (1,137 bp) was disrupted by replacing nucleotides 128–1,129 with a spectinomycin/streptomycin resistance cassette derived from pDW9 (1) in WT.
Δ4160	The *all4160* coding region (1,050 bp) was disrupted by replacing nucleotides 53–897 with a neomycin resistance cassette derived from pRL161 (2) in WT.
Δ*henR*/all4160	The plasmid pSU101 (3), carrying the upstream region (1,170 bp), coding sequence, and downstream region (42 bp) of *all4160*, was integrated into the all4160 locus of the Δ*henR* strain by homologous recombination via a single-crossover event.
Δ*henR*/S74A	Same as the Δ*henR*/all4160 strain, except that Ser74 of All4160 was substituted with Ala.
Δ2284	The *all2284* coding region (498 bp) was disrupted by replacing nucleotides 25–384 with a neomycin resistance cassette derived from pRL161 (2) in WT.
Δ3423	The *alr3423* coding region (444 bp) was disrupted by replacing nucleotides 6–380 with a neomycin resistance cassette derived from pRL161 (2) in WT.
Δ*henR*/Δ2284	The *all2284* gene was disrupted in the Δ*henR* strain.
Δ*henR*/Δ3423	The *alr3423* gene was disrupted in the Δ*henR* strain.

References

1. **Golden JW**, **Wiest DR**. 1988. Genome Rearrangement and Nitrogen Fixation in Anabaena Blocked by Inactivation of xisA Gene. Science **242**:1421–1423.

2. **Elhai J**, **Wolk CP**. 1988. A versatile class of positive-selection vectors based on the nonviability of palindrome-containing plasmids that allows cloning into long polylinkers. Gene **68**:119–138.

3. **Ehira S**, **Shimmori Y**, **Watanabe S**, **Kato H**, **Yoshikawa H**, **Ohmori M**. 2017. The nitrogen-regulated response regulator NrrA is a conserved regulator of glycogen catabolism in β-cyanobacteria. Microbiology **163**:1711–1719.

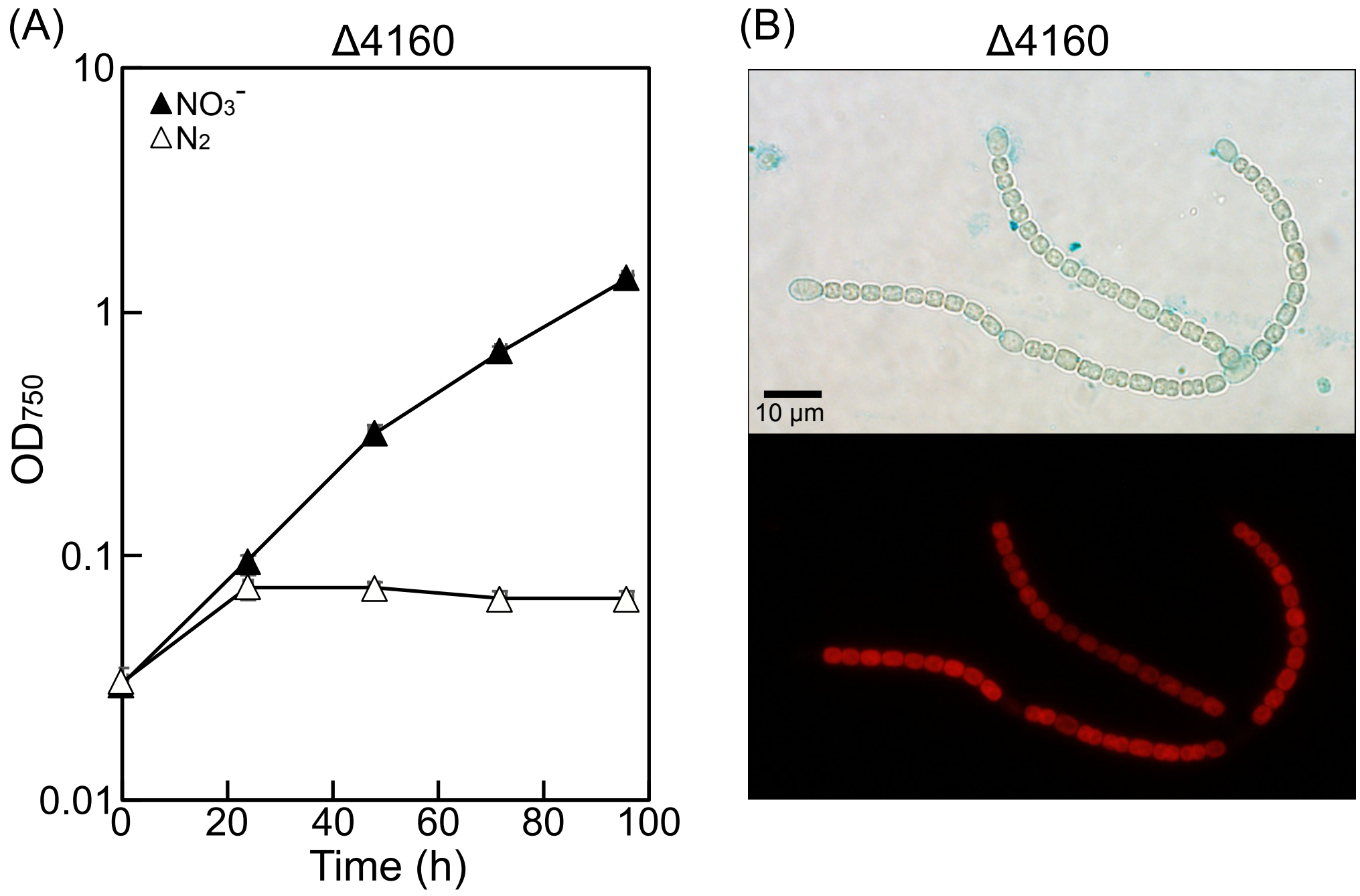

FigureS1. Phenotypes of the all4160 mutant (Δ4160)

(A) Growth of Δ4160 with nitrate (closed triangles) or dinitrogen (open triangles) as nitrogen sources.

(B) Micrographs of Alcian blue-stained Δ4160 cells. The upper panel shows bright-field images, and the lower panel shows autofluorescence.

```
RsbV    ---------------MNINVDVKQNENDIQVNIAGEIDVYSAPVLREKLVPLAEQG---ADLRI 46
SypA    ---------------MELHQFE-SNEDILVLSVQGDMDAIGCRDIQPSIDSVIEQEH--HQVQI 46
All4160 MYMATKVQSFMTSQPTEVNFLVTTLNETLIVQVPARLSVLEAVGFKQTCQELTKGNSHPKEIII 64

                   *
RsbV    CLKDVSYMDSTGLGVFVGTFKMVKKQGGSLKLENLSERLIRLFDITGLKDIIDISAKSEGGVQ 109
SypA    DLSHVAFLDSSGIGAIVYLYKRLIEKDRTMQIKNAHGQPLELLKLLRIENAIPVNKTTH---- 105
All4160 DFQQTTFMDSSGLGALVSNFKTTKEKGIVMILRNVTPQVMAVLNLTGLDKVFSIEPSSNIS-- 125
```

Figure S2. Multiple sequence alignment of STAS domain amino acid sequences.

A multiple alignment was generated using the STAS domains of RsbV from *Bacillus subtilis*, SypA from *Vibrio fischeri*, and All4160. Serine residues known to be phosphorylated in RsbV and SypA are indicated by an asterisk above the alignment (Yang *et al*., 1996; Morris and Visick, 2013). The corresponding residue in All4160 (Ser74) is conserved.

References

1. Morris AR, Visick KL. 2013. The response regulator SypE controls biofilm formation and colonization through phosphorylation of the syp-encoded regulator SypA in Vibrio fischeri. Mol. Microbiol. 87:509–25.

2. Yang X, Kang CM, Brody MS, Price CW. 1996. Opposing pairs of serine protein kinases and phosphatases transmit signals of environmental stress to activate a bacterial transcription factor. Genes Dev. 10:2265–2275.

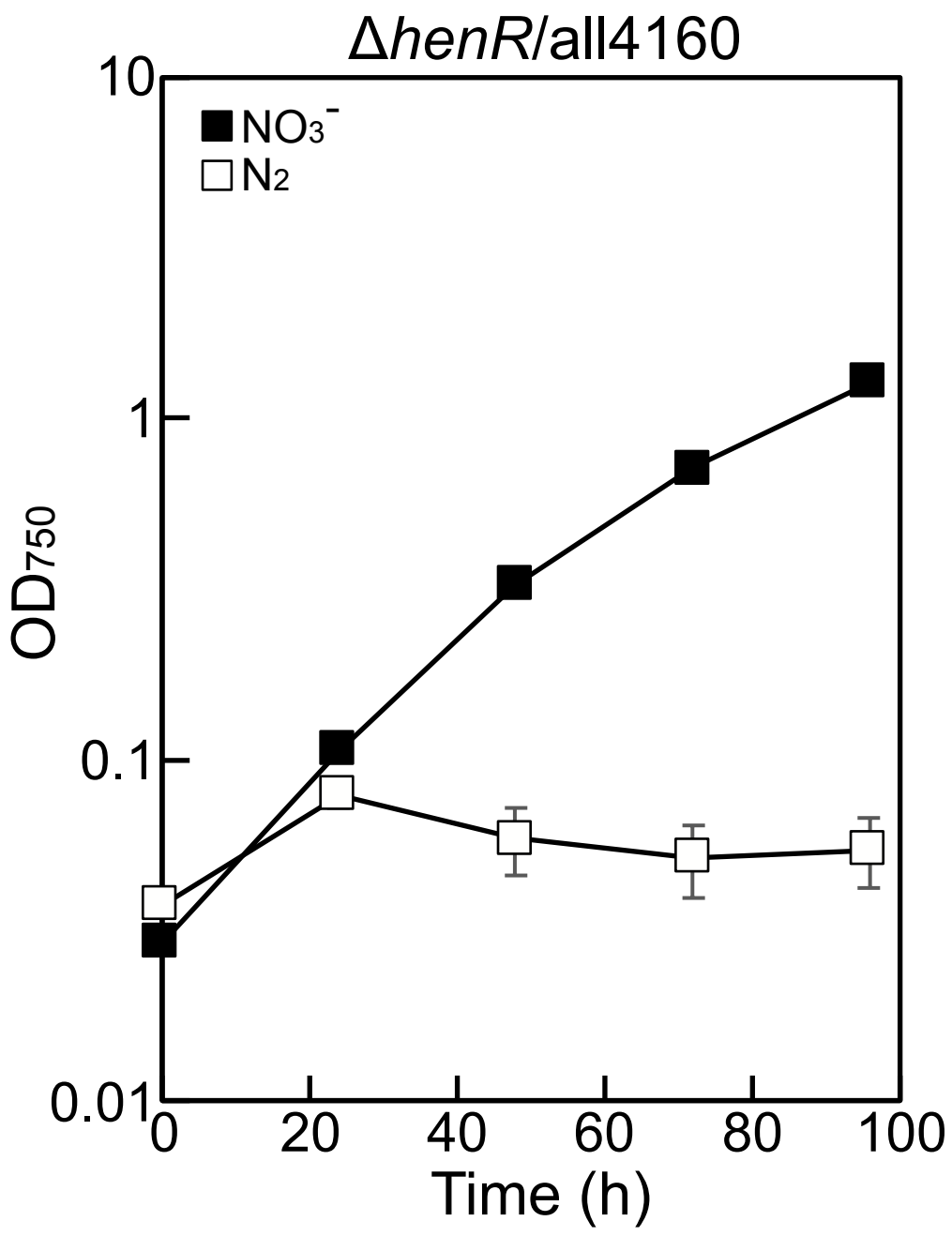

Figure S3. Expression of wild-type All4160 does not restore the nitrogen-fixation defect of Δ*henR*.

Growth of Δ*henR* carrying a plasmid expressing wild-type All4160 with nitrate (closed squares) or dinitrogen (open squares) as nitrogen sources.

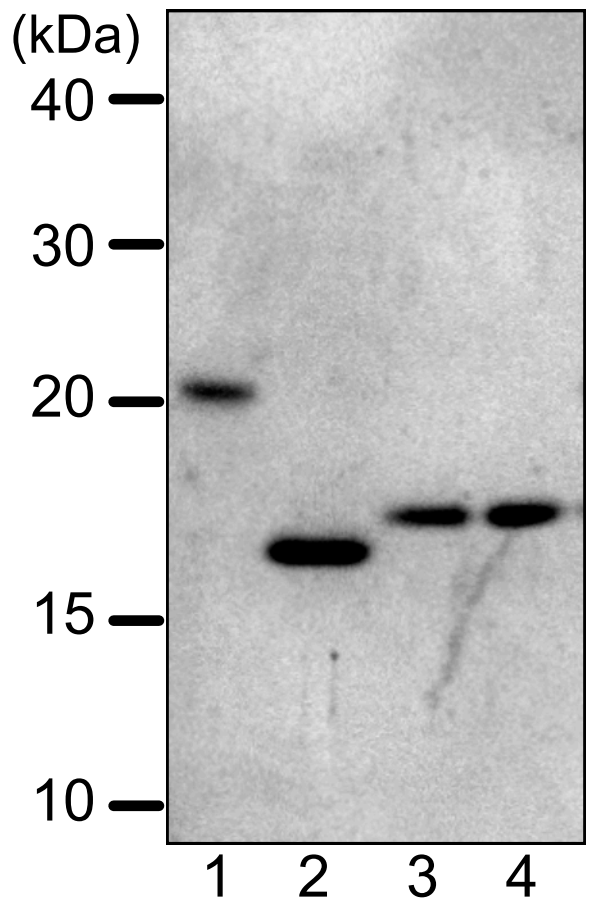

Figure S4. Recombinant proteins used for the phosphorylation assay.

Recombinant proteins used for the phosphorylation assay were analyzed by conventional SDS-PAGE. His-tagged recombinant proteins were expressed from the expression vector pColdII (Takara Bio) in *E. coli* BL21(DE3) and purified using TALON Metal Affinity Resin (Takara Bio). To generate a plasmid for the STAS domain of All4160, a DNA fragment encoding the STAS domain (residues 1-148) of All4160 was amplified and cloned between the NdeI and EcoRI sites of pColdII. Plasmids for All2284 and Alr3423 were constructed by cloning the coding regions into the EcoRI and SalI sites of pColdII. Proteins included in each lane were as follows: lane 1, All2284; lane 2, Alr3423; lane 3, the All4160 STAS domain; lane 4, the S74A variant of the STAS domain.
